# Morphological changes of glutamatergic synapses in animal models of Parkinson’s disease

**DOI:** 10.3389/fnana.2015.00117

**Published:** 2015-09-25

**Authors:** Rosa M. Villalba, Abraham Mathai, Yoland Smith

**Affiliations:** ^1^Yerkes National Primate Research Center, Emory UniversityAtlanta, GA, USA; ^2^UDALL Center of Excellence for Parkinson’s Disease, Emory UniversityAtlanta, GA, USA; ^3^Department of Neurology, Emory UniversityAtlanta, GA, USA

**Keywords:** Parkinson’s disease, striatum, subthalamic nucleus, synaptic plasticity, glutamatergic synapses, vGluT, astrocytes plasticity

## Abstract

The striatum and the subthalamic nucleus (STN) are the main entry doors for extrinsic inputs to reach the basal ganglia (BG) circuitry. The cerebral cortex, thalamus and brainstem are the key sources of glutamatergic inputs to these nuclei. There is anatomical, functional and neurochemical evidence that glutamatergic neurotransmission is altered in the striatum and STN of animal models of Parkinson’s disease (PD) and that these changes may contribute to aberrant network neuronal activity in the BG-thalamocortical circuitry. Postmortem studies of animal models and PD patients have revealed significant pathology of glutamatergic synapses, dendritic spines and microcircuits in the striatum of parkinsonians. More recent findings have also demonstrated a significant breakdown of the glutamatergic corticosubthalamic system in parkinsonian monkeys. In this review, we will discuss evidence for synaptic glutamatergic dysfunction and pathology of cortical and thalamic inputs to the striatum and STN in models of PD. The potential functional implication of these alterations on synaptic integration, processing and transmission of extrinsic information through the BG circuits will be considered. Finally, the significance of these pathological changes in the pathophysiology of motor and non-motor symptoms in PD will be examined.

## Basal Ganglia Nuclei and Connectivity

The basal ganglia (BG) are a collection of interconnected subcortical nuclei, including the striatum, globus pallidus (GP), substantia nigra, and subthalamic nucleus (STN), which closely interact with the cerebral cortex and thalamus. While historically considered as key components of the motor system, the BG receive cortical projections from all functional areas of the cerebral cortex and contribute to both motor and non-motor functions (Alexander et al., 1986; Mink, 1996). The information flow through the BG circuitry is segregated into motor, associative, and limbic/emotional domains based on their relationships with specific cortical projection areas and the engagement of these regions in various behaviors (Alexander et al., 1986; Lanciego et al., 2012). A large number of findings discussed in this review were gathered from the motor-related nuclei of the primate BG. The striatum, the major input structure of the BG, receives projections from the cerebral cortex, brainstem, and thalamus. The GP consists of two anatomically and functionally separate nuclei, the external and internal pallidal segments [GPe and GPi, respectively in primates; GP and entopeduncular nucleus (EPN) in rodents]. The substantia nigra also comprises two separate nuclei, the GABAergic pars reticulata (SNr) and the pars compacta (SNc), which contains pigmented dopamine (DA)-containing neurons. The dopaminergic neurons of the SNc project primarily to the striatum, but also provide significant innervation of other BG nuclei and the thalamus (particularly in primates; Smith and Kieval, 2000; García-Cabezas et al., 2009; Rommelfanger and Wichmann, 2010). The glutamatergic STN is a small nucleus which is intercalated between GPe and GPi. In addition to the striatum, the STN is also considered as a major entry for cortical information to the BG network (Nambu et al., 2000; DeLong and Wichmann, 2010), while the GPi (or EPN in non-primates) and SNr are the two main output nuclei of the BG.

The striatum and the STN receive topographically organized projections from functionally diverse regions of the cerebral cortex (Parent and Hazrati, 1995; Nambu et al., 1996). Because information flows more rapidly to the BG output nuclei via the corticosubthalamic projection than via the direct and indirect trans-striatal pathways, the trans-subthalamic route is commonly referred to as the “hyperdirect” pathway of the BG (Nambu et al., 2000, 2002; Sano et al., 2013; Smith and Wichmann, 2015). While the corticosubthalamic projection is less extensive than the corticostriatal system, it originates from motor, associative and limbic cortical regions and is a powerful source of excitation to STN neurons through which cortical inputs can rapidly regulate the activity of downstream BG output nuclei, the GPi and SNr (Monakow et al., 1978; Nambu et al., 2000; Haynes and Haber, 2013). Corticosubthalamic axons target dendritic spines and distal dendritic shafts of STN neurons in rats (Bevan et al., 1995; Mathai et al., 2015).

The GPe is another key structure of the BG which receives its main inputs from the striatum (the so-called indirect pathway) and the STN. In turn, two different populations of GPe neurons provide GABAergic innervation to all other BG nuclei. The so-called “arkypallidal” cells are the main sources of the pallidostriatal system, while the “prototypic” cells project massively to the STN, with collateral to the GPi and SNr (Shink et al., 1996; Smith et al., 1998a,b; Mallet et al., 2012; Dodson et al., 2015). There is recent evidence for a direct GABAergic/cholinergic pallidocortical projection in mouse (Saunders et al., 2015).

The BG outflow is directed at specific thalamic and brainstem nuclei via the GPi and SNr, largely through collateralized axonal projections (Parent and De Bellefeuille, 1982; Parent et al., 1983). The BG-receiving ventral motor thalamic nuclei project to widespread areas of the frontal lobe and send projections back to the striatum, while descending projections from the BG to the brainstem terminate massively in the pedunculopontine nucleus (PPN) which, in turn, provide significant ascending and descending projections to the thalamus, BG, reticular formation and spinal cord (Rye et al., 1988; Lavoie and Parent, 1994; Parent and Hazrati, 1995; Mena-Segovia et al., 2004). Recent evidence indicates that the descending trans-PPN projections may play an important role in regulating brainstem and spinal motor mechanisms related to gait and balance (Pahapill and Lozano, 2000; Garcia-Rill et al., 2011). The PPN is also part of several feedback circuits with projections to the BG and the thalamus (Rye et al., 1988; Lavoie and Parent, 1994; Mena-Segovia et al., 2004). Other projections from the SNr reach the superior colliculus, which is involved in coordinating head and eye movements, while a specific subset of peripallidal GPi neurons project massively to the lateral habenula, and play a role in the modulation of reward and limbic mechanisms (Wurtz and Hikosaka, 1986; Wickens, 2008; Hikosaka, 2010).

Recent studies suggest the existence of a direct glutamatergic cortico-pallidal projection in mammals, including humans (Mathai et al., 2012; Smith et al., 2014c; Milardi et al., 2015; Smith and Wichmann, 2015). This “cortico-pallidal” system is separate from the descending cortico-spinal and cortico-pontine axons that travel through the internal capsule (Naito and Kita, 1994; Milardi et al., 2015; Smith and Wichmann, 2015), and bypasses the traditional direct, indirect, and hyperdirect corticofugal pathways. The existence of this direct glutamatergic cortico-pallidal projection could have a significant impact on our present understanding of transmission and processing of information through the BG circuits in normal and diseased states (Smith and Wichmann, 2015).

## The Striatum: Main Entry to the BG Circuitry

The dorsal striatum, made up of the putamen and caudate nucleus in primates, is mainly innervated by sensorimotor (post-commissural putamen) and associative (caudate nucleus and pre-commissural putamen) cortices, respectively, while the ventral striatum (nucleus accumbens and olfactory tubercle) is the main target of limbic-related inputs from the hippocampus, amygdala and medial prefrontal cortices (Russchen et al., 1985; Alexander et al., 1986; McGeorge and Faull, 1987; Haber et al., 1995; Parent and Hazrati, 1995; Fudge et al., 2002). In the human literature the term “lenticular” or “lentiform” nucleus is commonly used to refer to the putamen and the GP (Carpenter and Sutin, 1983).

Each striatal region also receives prominent functionally-related thalamic inputs from intralaminar, relay, associative and midline nuclei. Among those, the caudal intralaminar nuclear group, the centre median (CM) and parafascicular complex (Pf), which innervates preferentially the putamen or caudate nucleus, respectively (Smith et al., 2004, 2009a; Galvan and Smith, 2011) is the predominant source of thalamostriatal projections. Massive dopaminergic innervation from either the SNc (to the dorsal striatum) or the ventral tegmental area (VTA; to the ventral striatum) provides key modulatory influences upon striatal processing of extrinsic cortical and thalamic information (Smith and Bolam, 1990; Nicola et al., 2000; Gerfen and Surmeier, 2011). Additional extrinsic inputs from the hypothalamus, GP, STN, raphe, locus coeruleus and PPN have also been described (Smith and Parent, 1986; Parent and Hazrati, 1995; Smith et al., 1998a,b; Ellender et al., 2011).

### Striatal Projection Neurons and Interneurons

The main targets of extrinsic inputs to the striatum are the GABAergic medium spiny neurons (MSNs), which represent 90–97% of all striatal neurons (Kemp and Powell, 1971a,b,c; Oorschot, 1996; Wickens et al., 2007a). These GABAergic neurons can be categorized into two main populations based on their hodological and chemical phenotypes. The “direct” pathway neurons send their main axonal projections directly to the output nuclei of the BG (i.e., GPi and SNr), and express preferentially the D1 DA receptors (D1R) and the neuropeptides substance P (SP) and dynorphin (DYN). On the other hand, the “indirect” pathway neurons project preferentially to the GPe, and express D2 receptors (D2R) and the neuropeptide enkephalin (ENK; Gerfen et al., 1990; Sidibé and Smith, 1999; Lanciego et al., 2004; Lei et al., 2004, 2013; Smith et al., 2009a, 2014a,b,c; Galvan and Smith, 2011; Gerfen and Surmeier, 2011; Huerta-Ocampo et al., 2014). Albeit less frequent, it is noteworthy that some striatal MSNs project to both GPe and GPi/SNr and co-express D1 and D2 DA receptor subtypes (Kawaguchi et al., 1990; Surmeier and Kitai, 1993; Surmeier et al., 1996; Wu et al., 2000).

The dendritic trees of both populations of striatal MSNs are covered with spines, which are the main targets of glutamatergic inputs from the cerebral cortex and thalamus. In rodents, the dendrites of individual MSNs harbor as many as 5000 spines (Wickens et al., 2007a). In addition to their glutamatergic innervation, striatal spines also receive synaptic inputs from midbrain dopaminergic neurons which frequently terminate onto the neck of the spine or a nearby segment of the dendritic shaft, thereby providing an anatomical substrate for close synaptic interactions between glutamatergic and dopaminergic inputs at the level of spines (Freund et al., 1984; Smith and Bolam, 1990; Smith et al., 1994, 2009a, 2014a; Nicola et al., 2000; Wickens et al., 2007a; Moss and Bolam, 2008). These functional interactions are critical for the development and maintenance of long-term synaptic plasticity of glutamatergic corticostriatal synapses (Nicola et al., 2000; Calabresi et al., 2007; Surmeier et al., 2007, 2010; Gerfen and Surmeier, 2011; Picconi et al., 2012). Although D1R and D2R MSNs display very similar morphological characteristics, the D2R MSNs exhibit increased excitability and harbor a less extensive dendritic tree than D1R cells in mice (Gertler et al., 2008; Kreitzer and Malenka, 2008; Fieblinger et al., 2014b), and each type of MSNs is differentially modulated by DA in normal and diseased states (Surmeier et al., 2007; Day et al., 2008; Kreitzer and Malenka, 2008; Shen et al., 2008; Kreitzer, 2009; Fieblinger et al., 2014b).

The aspiny interneurons are far fewer in number, accounting for about 3–10% of the total striatal population (Tepper and Bolam, 2004; Bernácer et al., 2005, 2007, 2012). Anatomically, they can be categorized into medium-sized GABAergic cells and large cholinergic neurons (Kawaguchi et al., 1995; Bernácer et al., 2007, 2012; Gonzales and Smith, 2015). Medium-sized GABAergic interneurons can be further classified histochemically into different subtypes: (a) parvalbumin-positive; (b) somatostatin-, neuropeptide Y-, and nitric oxide synthase-positive; (c) calretinin-positive (Tepper and Bolam, 2004; Bernácer et al., 2005, 2007, 2012); and (d) tyrosine hydroxylase (TH)-positive (Tepper et al., 2010). It is noteworthy that the latter subtype is rare in the normal primate striatum, but undergoes an upregulation after striatal DA denervation (Betarbet et al., 1997; Mazloom and Smith, 2006; Bernácer et al., 2012). It remains unclear if the various subtypes of TH-positive GABAergic cells described in TH-Cre mice (Tepper et al., 2010) represent the same neuronal phenotype as those seen in 1-methyl-4-phenyl-1,2,3,6-tetrahydropyridine (MPTP)-treated monkeys and Parkinson’s disease (PD) patients.

## DA Mesostriatal System

DA plays a fundamental role in normal BG function. The mesostriatal dopaminergic system, which comprises the mesolimbic and the nigrostriatal pathways, enables BG control of motor planning and action selection (Wurtz and Hikosaka, 1986; Berns and Sejnowski, 1998; Gurney et al., 2001). Because of its involvement in a wide array of physiologic and pathologic processes, the anatomical and functional organization of the DA mesostriatal systems has been the topic of extensive studies for many years (for reviews, see Wickens et al., 2007b,c; Kreitzer, 2009; Gerfen and Surmeier, 2011). Despite such interest, the exact role of DA in normal BG function is complex and remains poorly understood. The whole striatum is densely innervated by dopaminergic axons and terminals (Lavoie et al., 1989; Prensa and Parent, 2001; Matsuda et al., 2009; Bolam and Pissadaki, 2012; Pissadaki and Bolam, 2013) that originates from the ventral midbrain including the SNc (A9), VTA (A10) and retrorubral Area (RRA; A8). The A9 group is the most densely packed group of midbrain dopaminergic cells located in the SNc. Projections from SNc and RRA neurons terminate in the dorsal striatum, while VTA neurons are the main source of DA innervation to the ventral striatum (Gerfen et al., 1987; Lynd-Balta and Haber, 1994a, b). Dopaminergic terminal boutons represent nearly 10% of all striatal terminals (Groves et al., 1994). Like other monoamines, there is evidence that DA can mediate its effects in striatal and extrastriatal brain regions through neurotransmitter diffusion (Arbuthnott et al., 2000; Cragg and Rice, 2004; Arbuthnott and Wickens, 2007; Wickens et al., 2007b; Descarries et al., 2008; Moss and Bolam, 2008; Rice and Cragg, 2008; Rice et al., 2011). Consistent with this hypothesis, most DA receptors in the striatum are located extrasynaptically in spines and dendrites of striatal neurons (Hersch et al., 1995; Yung et al., 1995; Delle Donne et al., 1996, 1997; Nicola et al., 2000; Wang and Pickel, 2002; Gerfen and Surmeier, 2011).

### GABA and Glutamate: Co-transmitters of the Nigrostriatal System

Recent evidence indicates that DA neurons in the SNc and VTA are capable of co-releasing GABA with DA, and inhibit striatal projection neurons (Tritsch et al., 2012, 2014). It is estimated that 5–10% of SNc DA neurons express GAD65 and fewer than 1% contain the vesicular glutamate transporter 2 (vGluT2) in rodents (González-Hernández et al., 2001; Bérubé-Carrière et al., 2009; Hnasko et al., 2010). Therefore, distinct subpopulations of DA neurons may release GABA or glutamate, and reliable detection of IPSCs and EPSCs may result from innervation of SPNs by several DA neurons (Matsuda et al., 2009). Future studies are needed to better understand the physiological (or pathological) conditions under which GABA, glutamate and DA are released or co-released from nigrostriatal axons.

The release of GABA from DA terminals is independent of vesicular GABA transporter (vGAT), but requires activity of the vesicular monoamine transporter 2 (vMAT2) for vesicular loading. The inhibitory GABAergic synaptic transmission from DA neurons does not depend on synthesis of GABA by either GADs (GAD65, GAD67) or GABA transaminase, suggesting that DA neurons inhibit MSNs by releasing GABA they acquire from the extracellular space using membrane uptake of GABA (Tritsch et al., 2014). Although the actions of DA are not believed to be spatially localized (Arbuthnott and Wickens, 2007), this co-release of GABA may confer dopaminergic neurons an additional point-to-point mode of action, and the flexibility to differentially control GABAergic transmission in a target-dependent manner across their extensive axonal arbors (Tritsch et al., 2014). It is noteworthy that evidence for GABA expression and release from other populations of monoaminergic neurons has been reported in other brain regions (Iijima, 1993; Trottier et al., 2002; Maher and Westbrook, 2008; Hirasawa et al., 2009; Broadbelt et al., 2010). Together, these findings expand the repertoire of synaptic mechanisms available to monoaminergic cells, and suggest that perturbations of GABA co-transmission might contribute to the etiology of monoaminergic pathologies or to the therapeutic efficacy of vMAT2 antagonists in specific brain disorders.

There is also evidence that a certain contingent of SNc and VTA DA neurons can store and release glutamate via the vGluT2, providing an additional level of chemical heterogeneity to the nigrostriatal system (Sulzer et al., 1998; Chuhma et al., 2004; Bérubé-Carrière et al., 2009; Yamaguchi et al., 2013; Antal et al., 2014; Morales and Root, 2014; Trudeau et al., 2014). The localization of metabotropic glutamate receptor 5 at the edges of striatal dopaminergic synapses in the monkey striatum (Paquet and Smith, 2003) is consistent with these observations.

### Striatal DA Receptor Subtypes

In addition to the strong and segregated expression of D1R and D2R in direct and indirect pathway MSNs, both GABAergic and cholinergic interneurons also express different subtypes of DA receptors, and their activity is tightly regulated by DA, most particularly that of cholinergic interneurons, which express both D2R and D5R (Yan and Surmeier, 1997; Yan et al., 1997; Day et al., 2006; Wang et al., 2006; Surmeier et al., 2007; Kreitzer, 2009; Gerfen and Surmeier, 2011). D3R and D4R are also expressed in both the dorsal and ventral striata (Landwehrmeyer et al., 1993; Rivera et al., 2002; Centonze et al., 2003). DA receptors are expressed to variable degree in other BG nuclei, providing a substrate for extrastriatal DA functions (Smith and Kieval, 2000; Rommelfanger and Wichmann, 2010). In addition to their post-synaptic localization, DA receptors are localized pre-synaptically in glutamatergic and GABAergic terminals throughout the BG circuitry, providing multiple targets through which DA regulatory influences can impact neurotransmission in normal and diseased states. The readers are referred to comprehensive reviews of the topic for additional information (Arbuthnott et al., 2000; Reynolds and Wickens, 2002; Costa, 2007; Rice and Cragg, 2008; Surmeier et al., 2010; Gerfen and Surmeier, 2011; Rice et al., 2011).

## Glutamatergic Synaptic Plasticity in PD and its Models

### Striatal Spine Loss in PD

**Figure 1:**
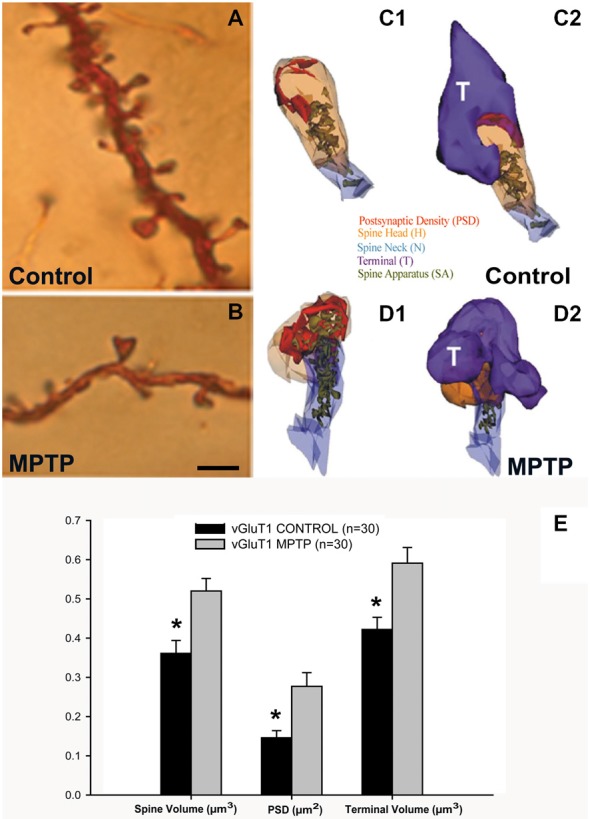
**Dendritic spines in the monkey striatum. (A,B)** Light micrographs of dendrites from Golgi-impregnated medium spiny neurons (MSNs) in the caudate nucleus of a control **(A)** and a 1-methyl-4-phenyl-1,2,3,6-tetrahydropyridine (MPTP)-treated **(B)** monkey. Note the dramatic spine loss of the dendrite of the MSN from the MPTP-treated monkey compared with control. **(C1–D2)** Three-dimension (3D)-reconstructed images of glutamatergic axo-spinous synapses from control **(C1,C2)** and MPTP-treated **(D1,D2)** monkeys. **(E)** Histogram comparing the morphometric measurements (mean ± SEM) for spine volume (μm^3^), post-synaptic density (PSD) area (μm^2^) and terminal volume (μm^3^) of structural elements at corticostriatal (vGluT1-positive) glutamatergic synapses using 3D reconstruction method of serial ultrathin sections collected from 30 axo-spinous synapses in each group from three control and three MPTP-treated animals. The spine volumes, the PSD areas, and the volume of vGluT1-containing terminals are significantly larger in MPTP-treated parkinsonian monkeys than in controls (*t-test; *p* < 0.001). Scale bar in **(B)** (applied to **A**) = 5 μm (See Villalba et al., 2009; Villalba and Smith, 2010, 2011a, 2013).

Striatal spine loss has been reported in the striatum of various animal models of PD and in parkinsonian patients. In both MPTP-treated monkeys and PD patients, the extent of spine pruning is tightly correlated with the extent of striatal dopaminergic denervation (Ingham et al., 1989; Stephens et al., 2005; Zaja-Milatovic et al., 2005; Smith and Villalba, 2008; Smith et al., 2009b; Villalba et al., 2009; Toy et al., 2014; Figures 1A,B, 4).

### Striatal Spine Loss on Direct vs. Indirect Striatofugal Neurons

Although there has been some controversy as to whether the striatal spine loss targets preferentially direct (D1R-positive) vs. indirect (D2R-positive) striatal MSNs, recent evidence indicates that both neuronal subtypes are affected, but through different mechanisms. Some authors reported that D2R striatopallidal neurons, but not D1R striatonigral neurons, selectively lose spines in reserpine-(systemic administration) and 6-hydroxydopamine (OHDA)-treated (injection in the medial forebrain bundle) mice with striatal DA depletion (Day et al., 2006). However, other reports described spine loss on both direct and indirect pathway neurons in intrastriatal 6-OHDA-treated or systemically MPTP-treated mice (Suárez et al., 2014; Toy et al., 2014). Similarly, both populations of striatal projection neurons undergo significant spine loss in monkeys chronically treated with low doses of MPTP (Villalba et al., 2009; Villalba and Smith, 2010, 2013). These findings are consistent with the homogeneous loss of spines across large populations of striatal MSNs described in Golgi studies of human parkinsonians and animal models of parkinsonism (Ingham et al., 1989; Stephens et al., 2005; Zaja-Milatovic et al., 2005; Smith and Villalba, 2008; Smith et al., 2009b; Villalba et al., 2009; Villalba and Smith, 2010, 2013). However, other monkey studies, using an acute regimen of MPTP toxicity, suggested a decrease in D2R spines accompanied with an increase in the density of D1R spines in the caudate nucleus of MPTP-treated cynomolgus monkeys (Scholz et al., 2008). The use of different animal models, different regimens and locations of neurotoxin administration, variable quantitative methods and observations of different striatal regions may contribute to these discrepancies.

In addition to spine pruning, recent evidence showed that the length and complexity of the dendritic tree of both direct and indirect pathway MSNs are significantly reduced in 6-OHDA-treated mice (Fieblinger et al., 2014a). In contrast to spine loss, that responds to L-DOPA therapy in this animal model, dendritic arbor atrophy is unresponsive to DA replacement therapy (Fieblinger et al., 2014a).

### Does Striatal Spine Loss Affect Corticostriatal and Thalamostriatal Synapses?

Using unbiased stereological synaptic counts, Ingham et al. (1998) reported ~20% decrease in the total number of axo-spinous asymmetric synapses in the striatum of 6-OHDA-treated rats. Recent findings from our laboratory also show a significant decrease in the total number of putative glutamatergic terminals (as revealed by asymmetric synaptic specializations) in the putamen of MPTP-treated parkinsonian monkeys (Villalba et al., 2013). To determine if this terminal loss is accounted for by a reduction in the number of cortical vs. thalamic boutons, antibodies raised against the vesicular glutamate transporter 1 (vGluT1) or vGluT2 were used as specific markers of corticostriatal or thalamostriatal terminals, respectively. Findings obtained in these studies remain controversial. On one hand, data from chronically MPTP-treated parkinsonian monkeys revealed that the relative density of vGluT1- or vGluT2-positive terminals in the putamen and the caudate nucleus is either unchanged or significantly increased compared with controls (Raju et al., 2008). These findings are consistent with human data showing a slight increase in the amount of vGluT1 protein expression in the putamen of PD patients compared with controls (Kashani et al., 2007). However, data from unilateral 6-OHDA-treated rats or mice indicate a profound reduction in the number of vGluT1-positive terminals, without any significant alteration in vGluT2-postive thalamic boutons, in these animals (Zhang et al., 2013; Fieblinger et al., 2014a). Whether these discrepancies are due to differences in the toxin being used (6-OHDA vs. MPTP), or the chronic nature of the MPTP regimen administered in monkeys compared with the acute 6-OHDA-induced lesion of the nigrostriatal projection in rodents remains to be determined.

Anatomical and functional data indicate that the loss of spines induces various forms of structural and functional synaptic homeostatic adaptations in MSNs of parkinsonian animals. For instance, the various components of vGluT1- and vGluT2-positive corticostriatal and thalamostriatal synapses undergo structural changes consistent with an increased synaptic strength, ie increase in the volume of the spines, increase in the size of the pre-synaptic terminals, increase in the area and complexity of the post-synaptic densities (PSD) and massive growth of the spine apparatus (Figures 1C–E), in the putamen of chronically MPTP-treated monkeys (Villalba and Smith, 2010, 2011a, 2013). Similar changes have been associated with intraspinous increase in protein synthesis and calcium buffering in other brain regions (Fifková et al., 1983; Bourne and Harris, 2008; Plotkin et al., 2013), thereby providing further evidence for increased corticostriatal glutamatergic transmission at these remaining synapses. However, this remains to be demonstrated using adequate electrophysiological approaches.

In a recent study, Fieblinger et al. (2014a) used glutamate uncaging approach at specific axo-spinous corticostriatal synapses, and found that the intrinsic excitability of direct pathway MSNs was increased, while that of indirect pathway neurons was decreased, in 6-OHDA-treated mice. On the other hand, the excitatory corticostriatal synaptic connectivity on indirect, but not direct, striatofugal neurons was lower in 6-OHDA-treated mice than controls. Finally, they also reported that in neither case was the strength of corticostriatal connections globally scaled (Fieblinger et al., 2014a). Together, these observations indicate that striatal MSNs undergo complex homeostatic (or pathologic) changes of glutamatergic synapses in response to striatal DA depletion that could affect differentially the direct and indirect striatofugal pathways in PD.

### Is the 6-OHDA-treated Rodent Model of PD Suitable to Study Striatal Spine Plasticity in PD?

Together, these recent findings (Zhang et al., 2013; Fieblinger et al., 2014a; Suárez et al., 2014) and previous studies (Ingham et al., 1989, 1998; Meshul et al., 2000; Day et al., 2006; Deutch et al., 2007; Neely et al., 2007) highlight the complex nature of the plastic changes striatal MSNs undergo in the 6-OHDA-treated rodent model of PD. However, the translation of these findings to the parkinsonsian state in humans must be achieved with caution because of the differential pathology of striatal glutamatergic afferents between the models under study and PD patients. Most importantly, PD is characterized by a massive degeneration of CM/Pf neurons (Henderson et al., 2000a,b; Smith et al., 2014a; Villalba et al., 2014), the main sources of the glutamatergic thalamostriatal system. The loss of these neurons and their corresponding axonal projections to the striatum is likely to further contribute to the synaptic homeostasis and scaling properties of remaining glutamatergic synapses in the PD striatum. Thus, the translation of morphological and functional studies of glutamatergic synapses in the striatum of PD models to the human parkinsonian condition must take into consideration the extent of CM/Pf degeneration (Villalba et al., 2013, 2014).

Although chronically MPTP-treated rhesus monkeys display 40–50% neuronal loss in CM/Pf (Villalba et al., 2013, 2014), the extent of Pf neuronal loss reported in various rodent models of PD is variable. While some authors did not find evidence for Pf degeneration 3 months after unilateral 6-OHDA nigrostriatal dopaminergic lesion in rats (Henderson et al., 2005; Kusnoor et al., 2012), other studies reported significant Pf cell loss in the same animal model (Aymerich et al., 2006; Sedaghat et al., 2009), or after systemic MPTP administration in mice (Freyaldenhoven et al., 1997). Some authors also showed that intrastriatal administration of 1-methyl-4-phenylpyridinium ion (MPP+) induces significant Pf cells damage in rats (Ghorayeb et al., 2002a). It remains to be determined whether these discrepancies were the result of differences in the neurotoxin exposure protocols, animal strains or other technical differences between these studies.

The need of animal models that include degeneration of the thalamostriatal system from CM/Pf is warranted for future studies of the plastic reorganization of striatal glutamatergic afferents in PD (Smith et al., 2014b; Villalba et al., 2014). Based on recent studies and others, it appears that MPTP toxicity might be a more reliable tool to induce CM/Pf neuronal loss and degeneration of the thalamostriatal system in mice and monkeys (Ghorayeb et al., 2002b; Smith et al., 2014a,b; Toy et al., 2014; Villalba et al., 2014).

### L-DOPA-induced Dyskinesias (LID) and Striatal Spine Plasticity

Although striatal spine loss has long been recognized in the striatum of DA-depleted animals and PD patients, the effects of DA replacement therapy on spine pruning, reorganization of synapic connectivity and homeostatic plasticity remains poorly understood. However, recent studies showed that L-DOPA therapy partly restores some structural and functional aspects of corticostriatal connection in rodent models of PD (Zhang et al., 2013; Nishijima et al., 2014; Suárez et al., 2014; Fieblinger and Cenci, 2015). Some authors, indeed, reported that the loss of spines and vGluT1-positive terminals in the striatum of 6-OHDA-treated rats and mice could be reversed by chronic treatment with L-DOPA (Zhang et al., 2013; Suárez et al., 2014). However, in animals that developed L-DOPA-induced dyskinesia (LID), the spines displayed abnormal synaptic relationships with vGluT1-positive terminals such that single spines often received synaptic inputs from 2 or more vGluT1-positive terminals (Zhang et al., 2013). Because this pathology was not found in non-dyskinetic L-DOPA-treated animals, the authors concluded that aberrant cortical innervation of striatal MSNs may be an important substrate of dysfunctional neuronal communication associated with LID (Zhang et al., 2013). Another main conclusion of this study was that neither the 6-OHDA lesion nor the L-DOPA treatment affected the prevalence and synaptic connections of vGluT2-positive thalamostriatal terminals in this animal model (Zhang et al., 2013). These observations were recently confirmed and extended in a recent study, which showed that both direct and indirect pathway MSNs manifest complex, and opposite, changes in homeostatic plasticity that affect their average firing rate in PD and LID states (Fieblinger et al., 2014a). Results of this study further demonstrated that the only adaptation found to be exclusively associated with LID was the restoration of excitatory axo-spinous synapses on the surface of indirect pathway neurons (Fieblinger et al., 2014a; see also Suárez et al., 2014).

As discussed above, an important shortcoming of these studies is the lack of evidence for thalamostriatal degeneration in the animal models used in these studies. The use of animal models of PD with CM/Pf pathology is essential to relate the neuroplastic properties of striatal MSNs and their glutamatergic responses to the human PD state (Smith et al., 2014b).

## Cellular, Molecular and Genetic Mechanisms for Striatal Spine Loss in PD

Although the mechanisms underlying striatal spine loss in PD remain unclear, there is converging evidence that intraspinous calcium (Ca^2+^) dysregulation likely contributes to this pathology (Segal et al., 2000; Sabatini et al., 2001; Oertner and Matus, 2005; Day et al., 2006; Deutch et al., 2007; Surmeier et al., 2007, 2011; Chen et al., 2008; Soderstrom et al., 2010; Surmeier and Schumacker, 2013). The Cav1.3α1 channels on D2R-containing neurons appear to be particularly important in mediating the spine pruning on indirect striatofugal neurons in mice (Day et al., 2006; Deutch et al., 2007; Surmeier et al., 2007; Soderstrom et al., 2010; Fieblinger et al., 2014b). In line with evidence that abnormal Ca^2+^ homeostasis may participate in this pathology, striatal MSNs devoid of the Ca^2+^ buffering protein, calbindin D-28k (CaB; Francois et al., 1994), such as those in the postcommissural putamen (sensorimotor striatal territory), display the most severe striatal spine pruning in parkinsonian monkeys (Smith and Villalba, 2008; Smith et al., 2009b; Villalba et al., 2009).

*In vitro* data suggest that the activation of the Ca^2+^-dependent protein phosphatase, calcineurin, and the up-regulation of the transcriptional activity of the myocyte enhancer factor 2 (MEF2) and related regulatory genes (Nurr77, Arc) participate in the loss of glutamatergic synapses and spines in the striatum (Pulipparacharuvil et al., 2008; Tian et al., 2010; Villalba and Smith, 2013). Evidence that cholinergic signaling through M1 muscarinic receptors and Kir2 potassium channels may trigger the loss of glutamatergic synapses in rodent models of parkinsonism has also been suggested (Shen et al., 2008). Because of its role in the regulation of neurite length and branching, LRRK2 mutation in PD may contribute to striatal spine pathology (MacLeod et al., 2006; Parisiadou et al., 2009; Lee et al., 2010).

## Changes in the Morphology of Astrocytes Associated with Glutamatergic Synapses in the Striatum of MPTP-Treated Monkeys

**Figure 2:**
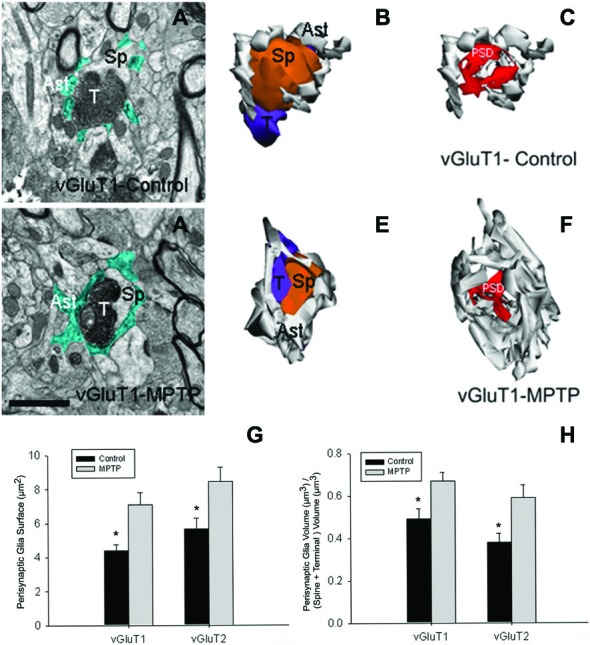
**Tripartite synapses (TS) in the monkey striatum. (A,D)** Electron micrographs of perisynaptic astrocytic processes (Ast) wrapping a vGluT1 axo-spinous synapse in control **(A)** and MPTP-treated **(D)** animal. **(B,C,E,F)** The three-dimensional (3D) reconstruction of a vGluT1-immunoreactive TS highlight the differences in the extensions of the astrocytic processes between control **(B,C)** and MPTP **(E,F)**. In the TS of control animals, the perimeters of the axon-spinous interfaces were only partially surrounded by astroglial processes **(B,C)**. In MPTP-treated animals, TS vGluT1-containing synapses displayed a large increase in astroglial processes ensheatment **(E,F)**. **(G)** Histograms comparing the surface area of perisynaptic glia associated with vGluT1- and vGluT2-immunopositive axo-spinous synapses in control (*N* = 3) and MPTP-treated (*N* = 3) monkeys (mean ± SEM). The surface of the perisynaptic glia was significant larger (*, *t*-test, *p* = 0.017 for vGluT1 and *p* = 0.006 for vGluT2) in MPTP-parkinsonian monkeys than in control. **(H)** Histograms comparing the ratio of the volume of the perisynaptic glia over the total volume of spine and the axon terminals in TS formed by vGluT1- or vGluT2-immunoreactive terminals. This ratio was significantly larger in MPTP than in control condition (*, *t*-test, *p* = 0.049 for vGluT1 and *p* = 0.028 for vGluT2). No significant difference was found between TS formed by vGluT1- or vGluT2-immunoreactive terminals. Total number of reconstructed spines = 32 (8 per group). Statistics were performed by using SigmaPlot (version 11.0). Abbreviations: Ast, astrocyte; PSD, post-synaptic density; Sp, dendritic spine; T, axon terminal (see Villalba and Smith, 2011b for details).

Data from our laboratory showed that in addition to the structural remodeling of the pre-synaptic terminals and post-synaptic spines at cortical and thalamic glutamatergic synapses (Villalba and Smith, 2010, 2011a, 2013), there is a significant growth in the extent of glial coverage of striatal glutamatergic synapses in parkinsonian monkeys (Villalba and Smith, 2011b; Figure 2). Perisynaptic astrocytes exhibit an interdigitated finger-like morphology in control animals (Figure 2A), while there is an expansion of astrocytic processes to cover a larger extent of the perimeter of axo-spinous complexes after MPTP-treatment (Figure 2). In MPTP-treated monkeys, the appositions between the axo-spinous complex and the astroglial processes are much tighter and continuous than in controls (Figures 2E,F). These differences between the normal and MPTP conditions were seen for both vGluT1- and vGluT2-positive glutamatergic synapses (Villalba and Smith, 2011b; Figures 2G,H).

A recent comparative study using 3D reconstruction in four animal models of PD, as well as in human PD, have shown that in response to DA denervation, astrocytes in both the striatum and GP occupy a larger striatal volume (Charron et al., 2014). This increase in striatal volume occupied by astrocytes in parkinsonism is due to an enlargement of astrocyte cell body and processes reorganization at the level of asymmetric synapses (Charron et al., 2014), but also to an increase in the number of astrocytes, a change known as reactive gliosis (Dervan et al., 2004; Henning et al., 2008; Charron et al., 2014). These morphological and ultrastructural changes in the perisynaptic astrocytes might underlie an active participation of glial processes in structural plasticity in the striatum, as previously shown in the hypothalamus (Theodosis et al., 2008) and hippocampus (Ventura and Harris, 1999; Witcher et al., 2007, 2010), suggesting that both glial and neuronal elements of axo-spinous glutamatergic synapses in the primate striatum are endowed with a high level of structural and functional plasticity. It is likely that such a synaptic arrangement is not homogeneous across all excitatory synapses (Ventura and Harris, 1999; Witcher et al., 2007, 2010), suggesting that some glutamatergic synapses may be more leaky and prone to spill over glutamate in the extracellular medium to activate extrasynaptic glutamate receptors than others.

These modifications in astrocytes morphology and in their spatial relationships with glutamatergic synapses in PD models, together with the different molecular mechanisms by which astrocytes respond to changes in neuronal activity, suggest that pathological changes in striatal astrocytes might play a key role in triggering and/or contributing to the morphological and functional changes in striatal network plasticity in parkinsonism (Villalba and Smith, 2011b). A better understanding of glia-neuronal communication in normal and pathological conditions might help to develop new PD neurotherapeutic strategies.

## Breakdown of the Corticosubthalamic Projection in Parkinsonism

**Figure 3:**
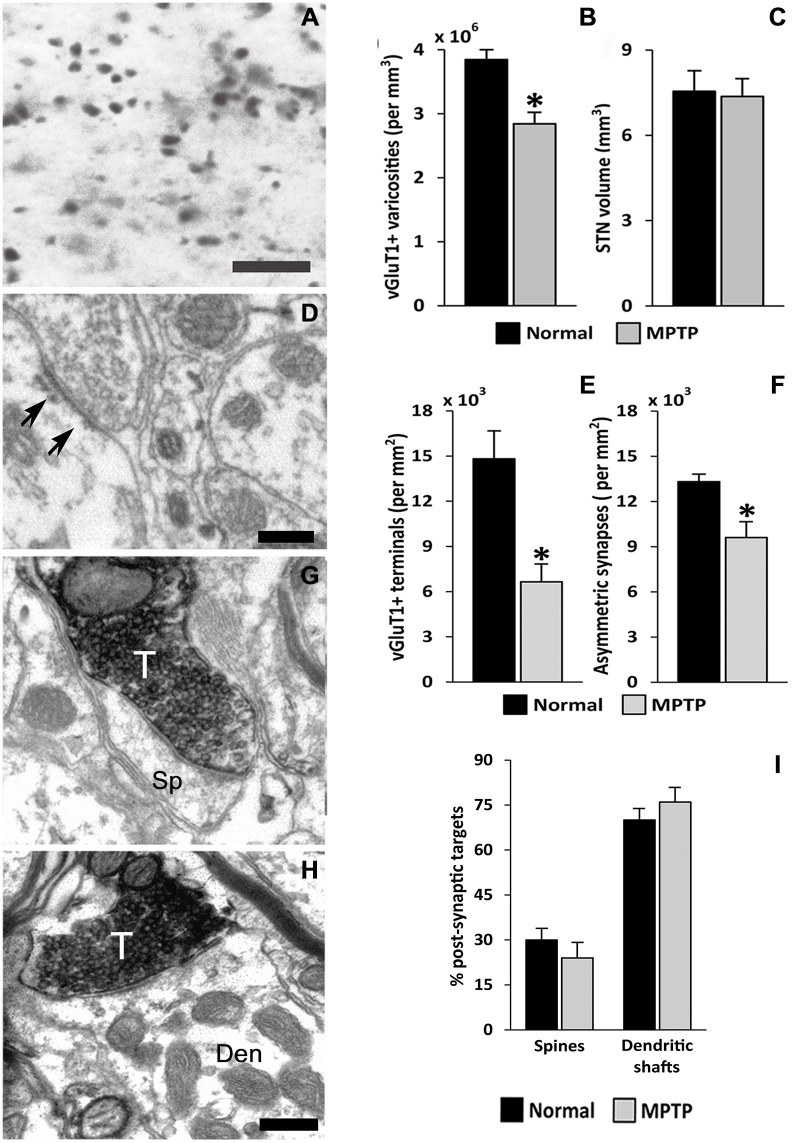
**vGluT1-positive innervation in the monkey subthalamic nucleus (STN). (A)** Light micrograph showing vGluT1-positive varicose processes. **(B)** Average density (mean ± SEM; *N* = 3) of vGluT1-immunoreactive varicosities in the dorsolateral STN of normal and parkinsonian monkeys (*, *t*-test, *p* = 0.012). **(C)** Comparison of the average STN volume (mean ± SEM; *N* = 3) between normal and parkinsonian monkeys. **(D)** Electron micrograph showing an asymmetric synapse (arrows) in the dorsolateral monkey STN. **(E)** Average density (mean ± SEM; *N* = 3) of vGluT1-immunopositive terminals in the dorsolateral STN of normal and parkinsonian monkeys (*, *t*-test, *p* = 0.02). **(F)** Average density (mean ± SEM; *N* = 3) of asymmetric synapses in the dorsolateral STN of normal and pakinsonian monkeys (*, *t*-test, *p* = 0.029). **(G,H)** Electron micrographs showing vGluT1-containing terminals forming asymmetric synapses with a spine **(G)** and a dendritic shaft **(H)**. **(I)** Post-synaptic targets of vGluT1-immunopositive terminals in the dorsolateral STN. No differences were found in the proportion of vGluT1-immunoreactive terminals forming asymmetric synapses with dendritic shafts and spines in normal and parkinsonian animals. Scale bar **A** = 10 μm and in (**D**; applies also to **G**) and **H** = 0.2 μm. Abbreviations: Den, dendrite; Sp, dendritic spine; T, axon terminal (See Mathai et al., 2015).

**Figure 4:**
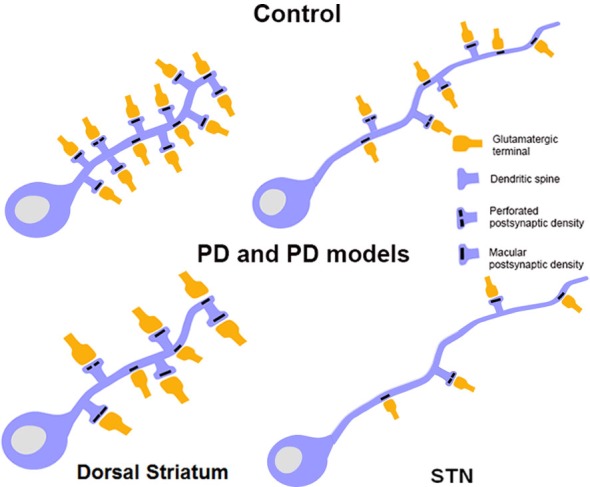
**Schematic showing morphological changes in dendritic spines and glutamatergic afferents in striatal MSNs and projection neurons in the STN in MPTP-treated parkinsonian monkeys.** In the striatum of parkinsonian monkeys, there is a significant reduction in the density of Sp on MSNs, but the remaining spines and terminals display an increase in volume. The size of the PSD at corticostriatal and thalamostriatal synapses is also increased and more commonly perforated in parkinsonian animals than controls. In the STN, there is an overall decrease in the prevalence of vGluT1-positive cortical terminals in contact with dendrites and spines of STN neurons in parkinsonian animals. Potential changes in the ultrastructure of spines and afferent glutamatergic terminals, as shown in the striatum, remain to be determined in the STN.

The striatum and the STN are the main entry points for cortical information to the BG. Glutamatergic inputs to the STN originate from the cerebral cortex (Monakow et al., 1978; Nambu et al., 1996; Haynes and Haber, 2013), the thalamus (Sadikot et al., 1992), the brainstem PPN (Lavoie and Parent, 1994) and local axon collaterals of STN neurons (Kita et al., 1983; Kita and Kita, 2012). The parkinsonian state is associated with ultrastructural remodeling of synaptic connections which may contribute to activity changes in the BG. So far, such changes have been documented for the corticostriatal, thalamostriatal and pallido-subthalamic projections (Ingham et al., 1989; Meshul et al., 2000; Villalba et al., 2009; Villalba and Smith, 2011a, 2013; Fan et al., 2012). In line with evidence that the activity of the hyperdirect corticosubthalamic projection is altered in PD (Mathai and Smith, 2011; Yamawaki et al., 2012; Shimamoto et al., 2013; de Hemptinne et al., 2013; Delaville et al., 2015), we found a significant breakdown of the corticosubthalamic projection, characterized by a profound loss of vGluT1-positive terminals in the STN of parkinsonian monkeys (Mathai et al., 2015; Figures 3, 4).

However, the functional impact of this pathology on the corticosubthalamic transmission and the downstream BG-thalamocortical circuitry remains to be clarified (Mathai et al., 2015). As shown in the striatum, possible homeostatic (or pathologic) changes in the strength and connectivity of remaining glutamatergic and GABAergic terminals in the STN might be induced (Ingham et al., 1989; Meshul et al., 2000; Smith et al., 2009b; Villalba and Smith, 2011a,b, 2013; Fieblinger et al., 2014a; Mathai et al., 2015). The known increase in the baseline (Bergman et al., 1994), and the greater degree of synchrony of STN neurons with cortical activity in PD are, indeed, in line with aberrant changes in corticosubthalamic transmission in the PD state (Williams et al., 2002, 2003, 2005; Moran et al., 2008; Gatev and Wichmann, 2009; Moshel et al., 2013; Shimamoto et al., 2013; Devergnas et al., 2014).

Thus, together with evidence for significant synaptic remodeling and altered glutamatergic transmission of the corticostriatal system in PD (Raju et al., 2008; Villalba and Smith, 2013; Fieblinger et al., 2014a), these findings suggest significant changes in the integration, processing and transmission of extrinsic cortical information to the BG in PD.

## Concluding Remarks

For the past 25 years, it has been well recognized that degeneration of the nigrostriatal DA system induces loss of spines and complex plastic changes in the anatomical and functional organization of glutamatergic synapses in the mammalian striatum (Figure 4; for a review, see Villalba and Smith, 2013). The loss of spines has been demonstrated in various animal models and confirmed in PD patients. It has also been shown that the extent of spine loss in the striatum is tightly correlated with the degree of striatal DA denervation, but not with the severity of parkinsonian motor features, at least in MPTP-treated monkeys (Zaja-Milatovic et al., 2005; Smith and Villalba, 2008; Smith et al., 2009b; Villalba et al., 2009). Controversies remain as to whether direct or indirect pathway neurons are preferentially affected by this spine pathology. The animal species and the toxin being used, the chronic vs. acute regimen of intoxication and the time points at which observations are being made post-lesion likely contribute to the variability of results obtained in recent years (Ingham et al., 1989, 1998; Stephens et al., 2005; Zaja-Milatovic et al., 2005; Day et al., 2006; Scholz et al., 2008; Villalba et al., 2009; Suárez et al., 2014; Toy et al., 2014). Although indirect pathway neurons appear to be more sensitive than direct pathway neurons at early time points after DA depletion induced by 6-OHDA or reversible DA depleting agent like reserpine (Day et al., 2006; Fieblinger et al., 2014a), chronic MPTP toxicity in non-human primates and mice models of PD induces more widespread pathological effects upon both populations of striatofugal neurons (Villalba et al., 2009; Toy et al., 2014). In addition to spine loss, it has become clear that striatal MSNs also undergo a significant reduction in the length and number of dendritic branches in rodent models of PD, and that such changes affect invariably both populations of striatofugal cells (Fieblinger et al., 2014a).

The impact of striatal spine loss on the anatomical and functional connectivity of cortical and thalamic glutamatergic afferents has also generated significant interest in recent years, but significant issues remain to be addressed. Although authors agree that striatal spine loss is associated with a decrease in the number of total striatal glutamatergic synapses in the striatum, controversy remains as to whether these are accounted for by the loss of cortical over thalamic synapses (Raju et al., 2008; Villalba et al., 2013; Zhang et al., 2013; Fieblinger et al., 2014a). In acute, 6-OHDA-treated animals, vGluT1-positive corticostriatal terminals are selectively affected, without any impact on thalamostriatal vGluT2-positive boutons (Zhang et al., 2013; Fieblinger et al., 2014a), while the total number of vGluT1-immunoreactive boutons and amount of vGluT1 protein expression in the striatum is not significantly affected in chronically MPTP-treated parkinsonian monkeys and PD patients (Kashani et al., 2007; Raju et al., 2008; Villalba et al., 2013). In regards to the impact of spine loss on the prevalence of thalamic terminals and synaptic organization of the thalamostriatal system, the situation remains unclear, and also appears to be affected by the animal model being used (Kashani et al., 2007; Raju et al., 2008; Villalba et al., 2013; Zhang et al., 2013; Fieblinger et al., 2014a).

The concerns raised in this review about the animal model being used to address issues related to glutamatergic plasticity in PD is particularly important in the case of the thalamostriatal system because of the differential extent of CM/Pf (or Pf in rodents) cell loss in various models of PD (Freyaldenhoven et al., 1997; Ghorayeb et al., 2002a; Henderson et al., 2005; Aymerich et al., 2006; Sedaghat et al., 2009; Kusnoor et al., 2012; Smith et al., 2014a,b; Villalba et al., 2014). The lack of information about CM/Pf cell loss in some rodent models used in previous studies of striatal synaptic plasticity is a major limiting factor that complicates the use of this model to assess neuroplastic properties of striatal neurons and glutamatergic afferents in relation to PD. Because CM/Pf neuronal loss is a key pathological feature of PD (Henderson et al., 2000a,b; Smith et al., 2014a; Villalba et al., 2014), combined with the fact that the CM/Pf is the main source of thalamic inputs to the striatum, we believe that studies of striatal glutamatergic systems plasticity must be achieved in animal models that display thalamic pathology (Toy et al., 2014; Villalba et al., 2014).

Another interesting issue that has been put forward in recent years in regards to striatal spine loss in PD is the fact that L-DOPA can restore the loss of spines on subsets (mainly D2R indirect pathway neurons) of striatal neurons in 6-OHDA-treated rats and mice. However, the chronic use of L-DOPA and the subsequent development of LID in this model is linked with the development of aberrant and excessive corticostriatal axo-dendritic and axo-spinous synapses (Zhang et al., 2013). It is unclear as to whether this pathological plasticity of the corticostriatal projection is also seen in other animal models of LID or in dyskinetic patients.

Although the striatum remains the BG structure that received most attention in studies of synaptic plasticity, recent evidence indicates that afferents to the STN are also morphologically and functionally disrupted in PD models. In MPTP-treated monkeys, a significant loss of vGluT1-containing cortical terminals has been reported, suggesting a partial degeneration of the hyperdirect corticosubthalamic pathway (Mathai et al., 2015). On the other hand, GABAergic GPe terminals also undergo major plastic changes that result in an increased strength of the pallidosubthalamic system in rodent models of PD (Fan et al., 2012). Ongoing studies are in progress to better understand the underlying mechanisms and the functional consequences of these plastic changes on the transmission, integration and processing of extrinsic information by STN neurons in PD.

## Conflict of Interest Statement

The authors declare that the research was conducted in the absence of any commercial or financial relationships that could be construed as a potential conflict of interest.
